# Trace of depression: Network structure of depressive symptoms in different clinical conditions

**DOI:** 10.1192/j.eurpsy.2022.12

**Published:** 2022-03-11

**Authors:** Satoshi Yokoyama, Go Okada, Koki Takagaki, Eri Itai, Kohei Kambara, Yuki Mitsuyama, Hotaka Shinzato, Yoshikazu Masuda, Ran Jinnin, Yasumasa Okamoto

**Affiliations:** 1 Department of Psychiatry and Neurosciences, Hiroshima University, Hiroshima, Japan; 2 Health Service Center, Hiroshima University, Hiroshima, Japan; 3 Graduate School of Humanities and Social Sciences, Hiroshima University, Hiroshima, Japan

**Keywords:** Beck Depression Inventory, depression, psychopathology network model

## Abstract

**Background:**

Psychopathological network model has received attention recently in the traditional debate about the continuity of depression. However, there is little evidence for comparing the network structure of depressive symptoms in several depressive states at different clinical stages. Through this study of a broad sample of patients with nonclinical to clinical depression, we examined differences in the network structure of depressive symptoms.

**Methods:**

Four groups of participants, including cohorts of clinical depression (current depression, *n* = 294; remitted depression, *n* = 118) and nonclinical depression (subthreshold depression, *N* = 184; healthy control, *n* = 257), responded to Beck Depression Inventory-II (BDI-II). After adjusting for age and sex, the residual scores of the 21 BDI-II items were input into a regularized partial correlation network for each group. Then, the estimated edge strengths/densities and node characteristics were compared.

**Results:**

Current depression has a discontinuous structure with a stronger and denser network of symptoms compared with nonclinical groups. Interestingly, remitted depression had improved to the level in healthy controls; however, it retained the same network structure as current depression, which indicates a trace of depression.

**Conclusions:**

We found the traces of depression that remained even after the symptoms disappeared. This study might provide a novel framework for elucidating the development and formation of depression.

## Introduction

Controversy often arises about the continuity of depression from a healthy state to a severe disease state. Several studies have demonstrated the existence of disease-specific boundaries. For example, depression-like analogues have been shown not to express some of the physical symptoms experienced by patients with depression [1]. Conversely, other studies indicating the continuity of depression have reported that factor structure differences between disease and healthy states are mostly attributable to differences in statistical methods [2]. A recent report using latent class analysis (LCA) partially supported each hypotheses, as follows: (a) there was a linear increase in the severity of most depressive symptoms, and (b) the severity of some symptoms shows a dramatic increase after exceeding a certain threshold [3]. Although the concept of a mental disorder spectrum is increasingly being considered, there remains debate regarding depression [4].

This lack of evidence stems from a framework of understanding based on the overall severity of depressive symptoms, that is, the sum of the scale scores. Patients with depression have heterogeneous symptom patterns. Patients with the same total score on depression questionnaires, including the Beck Depression Inventory-Second Edition (BDI-II) [5], often do not share a single symptom. A previous study examined the symptom profiles of 3,705 patients with depression based on combinations of 12 depressive symptoms [6]. The results indicated that patients could be discriminated into 1,030 unique symptom profiles, which further demonstrates that using the total score as a proxy for depression severity is unjustified. Accordingly, researchers have challenged the use of item scores of depression questionnaires to homogenize patients into smaller groups. Numerous factor analyses on the relationship among symptoms have categorized these symptoms as cognitive, emotional, and physical [7]. However, there are statistical criticisms of these results, as mentioned in the first paragraph. Additionally, the LCA results do not provide evidence for determining the dimensions or subtypes of depressive symptoms since they reflect the overall severity [8]. These analytical methods share the assumption regarding potential causes underlying the symptom items; however, such an assumption might not be warranted in psychopathological studies [9].

To move forward with this challenge, psychopathology network models [10] have been recommended for examining the interactions among symptoms [6, 11, 12]. Unlike traditional methods based on latent-variable models, network models treat individual depressive symptoms as important constructs. This allows the conceptualization of depression as a complex interaction resulting from the relationship among symptoms and their mutually vicious cycles [13]. Rather than categorizing symptoms into subtypes or latent factors, the network model demonstrates interactive paths and mediations among symptoms. Consequently, it can provide important evidence regarding whether cognitive symptoms of depression are mutually related, or that certain symptoms might be central to triggering others. A network model study on the longitudinal course of depression found that the network was more strongly connected in patients with persistent depression than in those with remitted depression, even after adjustment for overall severity [11]. Examining these symptom interactions, as well as clarifying the structural differences of depressive symptoms across different treatment states and clinical stages of depression, could facilitate elucidation of depression and prevention of worsening mental illness [14]. Moreover, network models allow objective assessment of symptom centrality [15], which identifies target symptoms to provide effective depression treatment and prevention [11, 16]. However, to our knowledge, there have been no studies on a network model of depressive symptoms based on the several continuous states reflecting the severity and clinical stage of depression.

Therefore, we aimed to use a network model to assess differences in the interrelations across depressive symptoms among patients with depression at clinically different states (current and remitted depression), populations with depression-like symptoms that do not meet the diagnosis of depression (subthreshold depression), and healthy controls. Comparisons among patient groups could help elucidate states associated with depressive symptoms. Further, comparisons between patients with remitted and nonclinical depression may facilitate the understanding of traits related to the depression diagnosis. Finally, comparing patients with current and nonclinical depression might inform research on the continuity of depression, including trait and state.

## Methods

### Study sample

Patients were diagnosed with major depressive disorder (MDD) by psychiatrists at Hiroshima University Hospital (*n* = 59) and nearby medical facilities, which included one psychiatric hospital (*n* = 47) and nine mental clinics (*n* = 306) in Hiroshima City. All included patients underwent a structured interview using the Hamilton Rating Scale for Depression (HAMD-17) [17], which was conducted by trained psychiatrists or clinical psychologists. As described by Frank [18], patients were grouped based on the score as follows: current depression (total HAMD-17 score ≥ 8; *n* = 294) or remitted depression (total HAMD-17 score ≤ 7; *n* = 118). Participants with subthreshold depression (*n* = 184) were derived from a previously studied cohort of subthreshold depression [19] and a previous randomized controlled trial [20]. Although they were different research projects from the patient group, both groups were clinically evaluated during the same period and participated in the study. The HAMD is designed to assess MDD severity and is unsuitable for assessing other depressive symptoms. Instead, subthreshold depression and healthy controls were classified based on the established BDI-II cut-off score. Although there are slight among-studies differences in the definition of subthreshold depression, we defined it as consecutive total BDI-II scores ≥ 13 on physical examination (week 0) and screening at study entry (week 20). This criterion score is defined in the BDI-II manual as the cut-off for minimal depression or worse [5]. None of the participants presented a major depressive episode; instead, they; showed chronic depressive symptoms (see previous studies for detailed eligibility criteria). Healthy controls (*n* = 257) were recruited from populations similar to those with clinical or subthreshold depression as the control group for group. Healthy controls had no history of psychiatric disorders, including MDD, and a BDI-II score ≤ 10. All the participants provided written informed consent for study participation and anonymous data collection. All research procedures were reviewed and approved by the Ethical Committees for Epidemiology and Clinical Research of Hiroshima University (approval numbers: E172-35, E-1513-4, E-566, and C-408).

Finally, we included 853 participants (current depression, *n* = 294; remitted depression, *n* = 118; subthreshold depression, *n* = 184; healthy control, *n* = 257).

### Beck Depression Inventory-II

The most widely used scale for assessing depression is the BDI-II (Japanese version by Kojima and Furukawa [21]), which is a self-report questionnaire consisting of 21 items that indicate depression severity. Each item is rated on a four-point Likert-scale of 0–3. The total score is 0–63, with higher scores indicating more severe depressive symptoms.

### Network estimation

We estimated a regularized partial correlation network with 21 nodes representing each BDI-II item. Four different network structures were generated through independent estimation for each group.

Before calculating the correlation matrices, which were the network inputs, we adjusted for the effects of age and sex on the BDI-II item scores. Subsequently, we estimated regularized partial correlation networks separately for the four groups using graphical LASSO [22]. Here, the edges represented the regularized partial correlation coefficients between two symptoms [12]. Our 21-node network required 210 edges for estimation, which was restricted by a small number of observations. LASSO [23], which is a regularization algorithm, was proposed to address this difficulty. Regularization is used to determine the optimal balance between parsimony and goodness-of-fit of the network; additionally, to avoid multiple testing problems arising in conventional significance testing [11]. We proposed a sparse (conservative) model because the regularization procedure pushed small connections to zero. We selected the best-fit network models using an extended Bayesian information criterion (EBIC). Regularized models using LASSO with EBIC have high specificity and varying sensitivity based on the sample size and true network structure [24].

Network models were visualized using the Fruchterman–Reingold algorithm (“spring” layout in the “qgraph” package). In this layout, we proximally placed highly correlated nodes. Regarding ease of viewing, we used the average layout of all the group samples. Blue and red edges represent positive and negative partial correlation coefficients, respectively. Nodes were colored based on the BDI-II subcategories [7] after being placed, which ensured that the node locations did not depend on the category type.

### Network comparison

First, we performed between-group comparisons of the frequencies of nonzero edges in the network. We applied a chi-square test using Holm’s correction method to verify the significance of the ratio differences. Subsequently, we performed a “Network Comparison Test” [25] for among-group comparisons of the network structure. This method involves a permutation test with 5,000 iterations of refitting to a randomly swapped dataset to assess between-network differences. The structural indicators for this comparison were global strength and network structure, which were quantified as the maximum differences in any edge weight. Statistical significance was set at 5%, Holm’s method was used to correct for multiple between-groups comparisons.

### Centrality measures

We calculated three centrality measures to assess the importance of nodes (items) in the network: Node strength [15] reflects how directly connected a node is to other nodes, that is, the sum of partial correlations between a node and all the other nodes. Closeness centrality [26] reflects how indirectly a node is connected to other nodes, that is, it is the inverse average of the shortest path lengths to all other nodes. Betweenness centrality [27] reflects the extent to which a node bridges two other nodes, that is, the percentage of node presence on all the shortest paths in the network. These three measures are often used to quantify node centrality [13, 15].

### Network accuracy and stability

To investigate the accuracy and stability of the edge strengths and the centralities, respectively, of the estimated network structures, we examined the accuracy of the edge weights based on a tutorial paper [13] using confidence intervals (CIs) with the bootstrapping method. Bootstrapping is a simple means of constructing CIs for complex statistics by repeating model estimation using sampled data. It is recommended to initially use the nonparametric bootstrap since the LASSO regularized network model biases the parametric bootstrap. We also used this method to estimate CIs and interpreted an edge as sufficiently strong if the bootstrapped CIs for the edge weights were nonzero. The number of bootstraps was set at 2,500.

Subsequently, to investigate the stability of centralities after only observing data portions, we used the case-dropping subset bootstrap, which applies the bootstrap to assess the correlation between the original centralities and those obtained from subsets in order to investigate the stability of centralities. Similar to an earlier report [13], the estimated centralities were considered stable if the correlation stability coefficient (CS-coefficient) was greater than 0.25.

### Software for network analysis

A series of network analyses were undertaken using R ver. 3.6.1 according to previous tutorial papers [13, 28]. The analysis packages and codes have been previously described in the cookbook (http://sachaepskamp.com/files/Cookbook.html) and are available on the internet. Graphical LASSO with EBIC was automatically estimated using the “EBICglasso” function of the “qgraph” package [29]. Among-network comparisons were performed using the “network comparison test” package [25]. To assess stability, we evaluated the CIs of the three centralities by the bootstrapping using the “bootnet” package [28].

## Results

### Comparison of scores per item

Table 1 shows the residual score for each BDI-II item in each group. The residuals were obtained by adjusting for covariates before computing the input matrix of the network. Note that the residuals are listed in Table 1 since they underwent significance tests. The original BDI-II scores are shown in parentheses below each residual score for a general overview of the severity of each group. There were significant group differences for all BDI-II items, with current depression having the highest score, with subthreshold depression following or having a similar score for all the items. Remitted depression was equal to, sometimes below, those of the healthy controls.

**Table 1.** Results of analysis of the residual score for each BDI-II item.

		Current depression^a^		Remitted depression^b^		Subthrehold depression^c^		Healthy control^d^		
		Mean	SD	Mean	SD	Mean	SD	Mean	SD	
1	Sadness	0.58	0.77	−0.72	0.62	0.16	0.58	−0.45	0.48	a > c > d > b
		(1.66)	(0.75)	(0.50)	(0.58)	(0.89)	(0.59)	(0.30)	(0.47)	
2	Pessimism	0.51	0.75	−0.63	0.67	0.31	0.84	−0.51	0.51	a > c > b = d
		(1.59)	(0.73)	(0.59)	(0.67)	(1.04)	(0.85)	(0.23)	(0.50)	
3	Past failure	0.41	0.90	−0.38	0.73	0.30	0.72	−0.51	0.50	a = c > b > d
		(1.44)	(0.88)	(0.75)	(0.69)	(1.13)	(0.73)	(0.32)	(0.50)	
4	Loss of pleasure	0.57	0.81	−0.66	0.72	0.01	0.60	−0.36	0.35	a > c > d > b
		(1.57)	(0.80)	(0.56)	(0.63)	(0.47)	(0.61)	(0.12)	(0.33)	
5	Guilty feelings	0.40	0.79	−0.44	0.51	0.16	0.67	−0.37	0.40	a > c > b = d
		(1.11)	(0.78)	(0.34)	(0.49)	(0.70)	(0.68)	(0.18)	(0.39)	
6	Punishment feelings	0.46	1.07	−0.58	0.70	0.14	0.78	−0.36	0.26	a > c > b = d
		(1.19)	(1.07)	(0.30)	(0.66)	(0.54)	(0.79)	(0.05)	(0.24)	
7	Self-dislike	0.62	0.95	−0.72	0.71	0.31	0.97	−0.61	0.37	a > c > b = d
		(1.67)	(0.91)	(0.45)	(0.67)	(1.04)	(0.98)	(0.13)	(0.36)	
8	Self-criticalness	0.63	0.94	−0.73	0.68	0.18	0.90	−0.51	0.39	a > c > b = d
		(1.63)	(0.90)	(0.41)	(0.66)	(0.83)	(0.91)	(0.15)	(0.38)	
9	Suicidal thoughts or wishes	0.35	0.76	−0.39	0.43	0.06	0.56	−0.26	0.25	a > c > b = d
		(0.86)	(0.75)	(0.19)	(0.40)	(0.37)	(0.58)	(0.06)	(0.24)	
10	Crying	0.42	0.94	−0.55	0.65	0.03	0.68	−0.26	0.43	a > c > d > b
		(1.23)	(0.94)	(0.38)	(0.64)	(0.51)	(0.70)	(0.23)	(0.43)	
11	Agitation	0.47	0.79	−0.53	0.56	0.05	0.62	−0.33	0.23	a > c > d > b
		(1.17)	(0.78)	(0.31)	(0.50)	(0.41)	(0.62)	(0.05)	(0.21)	
12	Loss of interest	0.53	0.87	−0.66	0.64	0.11	0.65	−0.38	0.32	a > c > d > b
		(1.43)	(0.86)	(0.41)	(0.59)	(0.58)	(0.67)	(0.11)	(0.31)	
13	Indecisiveness	0.57	0.89	−0.68	0.65	0.07	0.78	−0.39	0.35	a > c > d > b
		(1.50)	(0.86)	(0.43)	(0.61)	(0.54)	(0.79)	(0.11)	(0.35)	
14	Worthlessness	0.55	0.89	−0.63	0.67	0.37	0.93	−0.61	0.38	a = c > b = d
		(1.50)	(0.87)	(0.42)	(0.64)	(1.09)	(0.94)	(0.11)	(0.36)	
15	Loss of energy	0.58	0.68	−0.67	0.64	0.11	0.60	−0.43	0.41	a > c > d > b
		(1.65)	(0.64)	(0.58)	(0.60)	(0.71)	(0.61)	(0.19)	(0.41)	
16	Changes in sleeping pattern	0.43	0.97	−0.60	0.62	0.06	0.62	−0.26	0.61	a > c > d > b
		(1.50)	(0.97)	(0.51)	(0.61)	(1.05)	(0.62)	(0.73)	(0.60)	
17	Irritability	0.40	0.97	−0.50	0.46	0.05	0.62	−0.27	0.21	a > c > d > b
		(0.99)	(0.97)	(0.20)	(0.40)	(0.35)	(0.63)	(0.05)	(0.21)	
18	Changes in appetite	0.41	0.85	−0.56	0.50	0.09	0.76	−0.28	0.56	a > c > d > b
		(1.23)	(0.85)	(0.31)	(0.50)	(0.76)	(0.77)	(0.39)	(0.56)	
19	Concentration difficulty	0.55	0.75	−0.72	0.58	0.18	0.72	−0.43	0.48	a > c > d > b
		(1.62)	(0.73)	(0.47)	(0.55)	(0.93)	(0.73)	(0.34)	(0.47)	
20	Tiredness or fatigue	0.53	0.81	−0.65	0.63	0.10	0.54	−0.39	0.42	a > c > d > b
		(1.62)	(0.79)	(0.62)	(0.60)	(0.71)	(0.55)	(0.24)	(0.43)	
21	Loss of interest in sex	0.40	0.99	−0.56	0.93	0.01	0.61	−0.20	0.36	a > c > d > b
		(1.56)	(1.06)	(0.94)	(1.06)	(0.29)	(0.63)	(0.11)	(0.33)	

*Note:* Residual values were obtained after adjusting for age and sex. Positive and negative valences are added since they present the difference between the values predicted by these covariates. The original score for each item may facilitate an intuitive understanding. Raw scores of the BDI-II are denote in parentheses below each residual score. The means of the item residual scores were tested using multivariate analysis of variance using the among-item covariance structure. The last table column shows the test results (all *p <* 0.001).

Abbreviation: BDI-II, Beck Depression Inventory-II.

### Regularized partial correlation network per group

**Figure 1.**
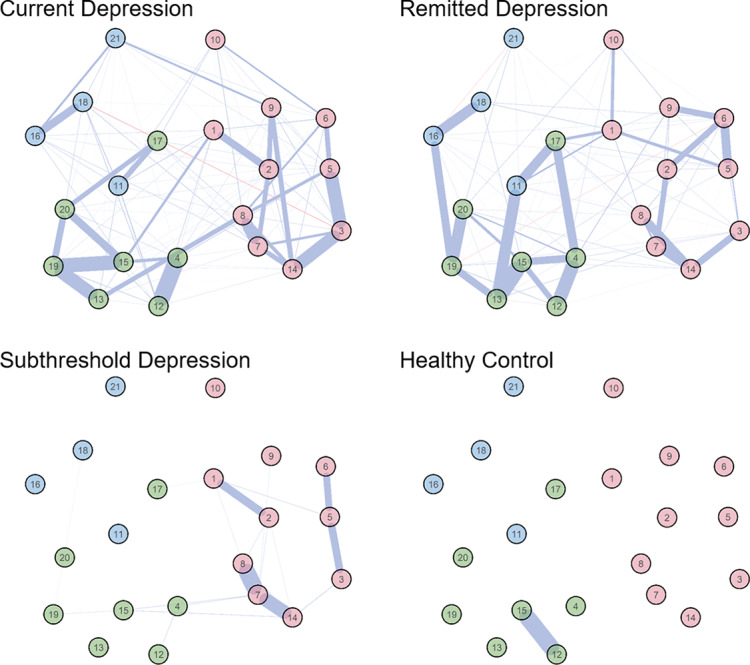
Network structures of the Beck Depression Inventory-II (BDI-II) for each group sample. The node number corresponds to the item number in the BDI-II. The edge thickness is proportional to the absolute value of the regularized partial correlation. These values were estimated from different samples for each group. Node colors correspond to the symptom categories in a three-factor model [7]: negative attitude (pink), performance difficulty (green), and somatic elements (blue). Positive and negative connections are represented by blue and red edges, respectively. There were no edges between these nodes since regularization shrinks small edges to zero. The network was drawn by placing highly correlated nodes close together. A group average layout was used to facilitate visual comparison.

Figure 1 shows the estimated network structures of BDI-II for each group. The network was mostly positively connected. We observed the strongest connections within the same symptom category in each network. For example, in the current depression network, the strongest connections occurred found between “Past Failure” (item 3) and “Guilty Feelings” (item 5) or “Worthlessness” (item 14) in the negative attitude category, as well as between “Loss of Pleasure” (item 4) and “Loss of Interest” (item 12), and between “Loss of Energy” (item 15) and “Concentration Difficulty” (item 19) in the performance difficulty category.

### Node bridging symptom categories

The nodes that bridged the symptom categories between the patient groups differed in some cases. For current depression, “Self-Criticalness” (item 8) and “Indecisiveness” (item 13), as well as “Sadness” (items 1) and “Loss of Energy” (item 15) were connected with negative attitude (pink color) and performance difficulty (green color). Contrastingly, for remitted depression, “Sadness” (items 1) and “Irritability” (item 17) were connected with both categories. Regarding the edges linking performance difficulty to somatic elements (blue color), in addition to edges common to current depression (“Irritability” [item 17] and “Agitation” [item 11]), other nodes strongly connected those categories in remitted depression (edges between “Indecisiveness” [item 13] and “Agitation” [item 11], as well as between “Concentration Difficulty” [item 19] and “Changes in Sleeping Pattern” [item 16]). There was a significant connection between “Suicidal thoughts or Wishes” (item 9) and “Loss of Interest in Sex” (item 21), only for current depression.

### Comparison of the number of connections in the network

The patient groups had frequency connections between symptoms (nonzero edges: current depression = 119, remitted depression = 106). Additionally, the nonclinical groups had sparse network structures (nonzero edges: subthreshold depression = 20, healthy control = 1). Chi-square tests for the ratio of the number of nonzero edges revealed significant differences, except between patient groups (Holm’s correction of multiple comparisons for significance level: Current depression = Remitted depression > Subthreshold depression > Healthy controls, all corrected *p* < 0.001). As shown in Table 1, remitted depression maintained the associations between symptoms even with improvement in the severity of most symptoms. Specifically, the symptom network structure of patients with depression was preserved independent of the clinical state.

### Comparison of the connection strengths in the network

Network comparison test indicated significant differences in global strength between current depression and subthreshold depression (*p* < 0.0001), current depression and healthy controls (*p* < 0.0001), and remitted depression and healthy controls (*p* < 0.0001). However, there was no significant difference between remitted depression and current depression (*p* = 0.62) or subthreshold depression (*p* = 0.12). There was a significant difference in network structure only between current depression and subthreshold depression/healthy controls (*p* < 0.0001 for both).

### Edge strength, closeness, and betweenness centralities

**Figure 2.**
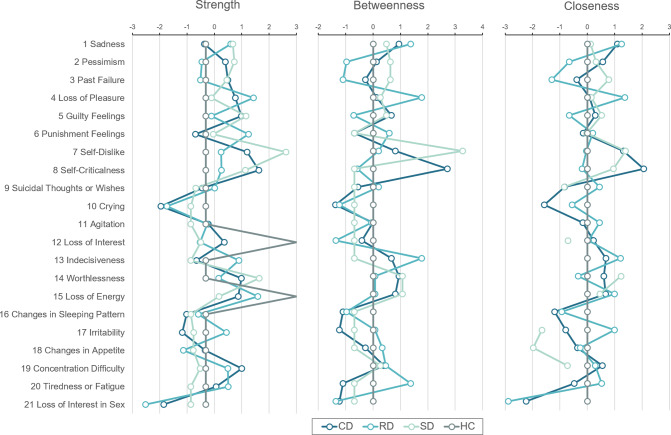
Standardized node centralities (Strength, Closeness, and Betweenness) for the Beck Depression Inventory-II (BDI-II) symptoms in each group. The centrality values for BDI-II items are shown as standardized *Z*-scores. The line color distinguishes current depression (CD), remitted depression (RD), subthreshold depression (SD), and healthy controls (HC).

Figure 2 shows the standardized node centralities of the respective BDI-II items. Current depression showed high centralities in “Self-Dislike” (item 7) and “Self-Criticalness” (item 8), with item 8 especially high in betweenness centrality. In this group’s network, item 8 was not only strongly associated with other symptoms in the negative attitude category but also formed a bridge with the performance difficulty category. This indicated that this symptom might be central to the variability in many depression symptoms. Similarly, item 7 showed that the three centrality indicators were especially high for subthreshold depression. Self-Disappointment was strongly associated with other depressive symptoms and is centrally involved in their depression network. Item 8 showed highly strength and closeness. However, apart from current depression, it did not mediate other symptoms. Although it is associated with other symptoms, it is unlikely to amplify the other symptoms by itself. This could have occurred via item 7, which is associated with item 8. Remitted depression showed differences in closeness and betweenness compared with the other groups. Pessimistic symptoms, including regret and blame for past selves, were isolated; however, there was high centralization of several symptoms in the performance difficulty category, including deficits in pleasure or decision-making and fatigue (items 4, 13, and 20).

### Stabilities of connectivity strength and centralities

**Figure 3.**
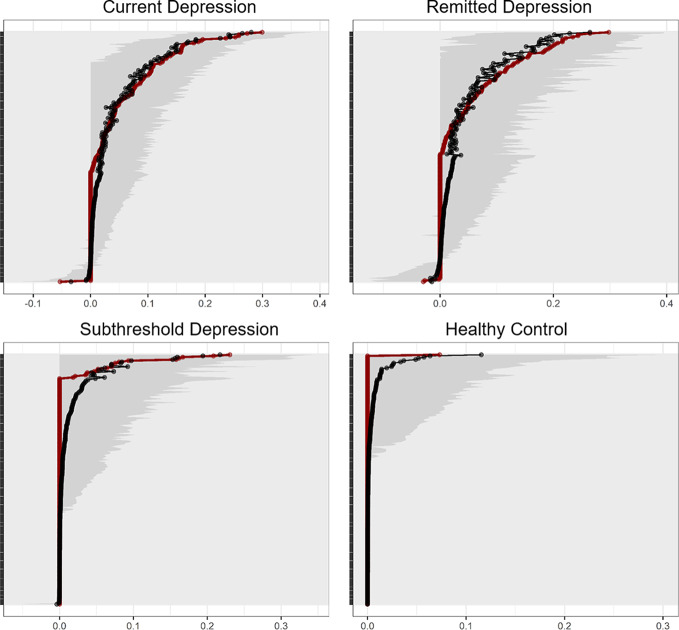
Bootstrapped confidence intervals of estimated edge weights for the estimated networks. The red lines represent the sample values. The black line shows bootstrapped means. The gray areas show bootstrapped confidence intervals (CIs). The *y*-axis in each graph represents each network edge according to edge weight in descending order.

Figure 3 shows the edge strengths estimated from the sample and bootstrapped CIs. The estimated strengths and bootstrapped means were similar for each edge. None of the bootstrap CIs straddled to zero on the edges where the absolute value of the calculated strength from the sample (red) was greater than zero. For some edges, the bootstrapped means slide more positively than the estimates from the sample in subthreshold depression and healthy controls. Although the estimated networks were overly sparse compared to bootstrapping, they might successfully exclude pretense/small associations in the network. Although this is a general advantage of regularization, our conservative estimation prevented verification that unconnected nodes are really unconnected. The CS-coefficient, which is based on the maximum percentage of cases that can be dropped out to maintain the estimated centrality, reached the previously reported cut-off value (>0.25) [13] only for the strength of current depression. Therefore, the findings regarding centralities should be undertaken with caution.

## Discussion

This study assessed differences in the interrelationships between depressive symptoms using a network model for three clinical depression states and healthy controls. Our findings provided a better understanding of the continuity of depression; moreover, they demonstrated that traces of depression persist even after the symptoms disappeared.

### Overall findings: Partial continuity of structural depression features and connection strength

Our findings showed that high network density (number of connections) might be a depression trait, and that the strength of among-symptom connections partially reflects the symptom status. Negatively biased thoughts and self-related feelings are characteristic factors of cognitive vulnerability in depression [30, 31]. We observed a gradual decrease in the connections within the negative attitude category from current depression to subthreshold depression. The maturation of associations among the symptom clusters, and their increasing closeness to other symptoms clusters, including somatic elements, determine depression. However, performance difficulties may be more closely related to symptom relapse than to cognitive vulnerability in remitted depression. Consequently, although the network structure of depressive symptoms partially revealed a continuum from patients with subthreshold depression who have high depressive symptoms to patients with depression who have remitted symptoms, patient with current depression showed a discontinuous symptom structure. Our findings are consistent with the findings of a recent LCA that revealed a linearly increasing, but nonlinear change, in the severity of symptoms [3].

### Valuable findings: Discontinuity of network density as the trace of depression

Notable, current and remitted depression showed almost identical network structures, regardless of severity. Further, remitted depression and healthy controls showed a similar degree of improvements in the individual scores for symptoms. Therefore, the association between the symptoms remained, just as the trace of depression, even after remission.

The dense network structure explains the tendency of multiple symptoms to simultaneously occur in depression. Individuals who have once experienced depression have negative memory and cognitive biases that disappear after remission, they can be easily reactivated by minor negative stimuli [32]. Based on the Differential Activation Hypothesis [32], patients who have experienced a major depressive episode form an internal coalition of mood, thought, and memory. Therefore, the processing patterns activated by a minor negative mood in these individuals differ from those in healthy controls. Actually, our results showed differences in the symptom network structure between patients with remitted depression and healthy controls. Susceptibility to depression recurrence may be attributed to the individuals having a dense network that is different from that in nonclinical subjects. Patients with remitted depression present with residual symptoms [33]. However, in clinical observations, we should carefully consider both symptom scores and the maintenance of among-symptom associations. Because the described traces of depression are difficult to detect using traditional approaches based on simple scores, our approach has clinically meaningful benefits. Furthermore, the efficacy of several depression treatments has been assessed based on the disappearance of the overall number of symptoms rather than the individual associations of depressive symptoms. Further studies targeting this trace dense network are warranted to evaluate treatment effects. This could prompt the reevaluation of conventional treatments and the development of new treatments.

### Reproducibility of the findings in our network

A previous network study described that current depression has a more closely connected network than remitted depression [11]. In this previous study, fatigue and guilty feelings were found to be more important for current depression than for remitted depression. These differences may be explained by a network approach based on partial correlations at different scales. This earlier study selected 16 items from the 30-item self-reported Inventory of Depressive Symptomatology, which were aggregated into 11 nodes. Contrastingly, the BDI-II has numerous cognitive symptoms and similar symptom items, including Item 4 (loss of pleasure) and Item 12 (loss of interest). These scale differences could influence the reproducibility of network analysis. In fact, a depression network using the BDI-II [16] found a negative cognitive community similar to ours. In the two-factor model of the BDI-II [5], item 9, “Suicidal Thoughts or Wishes”, represents the cognitive dimension. In our current depression network, item 9 was well-connected to these cognitive items. Therefore, our findings are consistent with those of previous approaches using the same scale. What differs from previous factorization approaches using the BDI-II is that our network detected edges connecting different factor categories. The edge between items 8 and 13, which were strongly connected in current depression, reflects the assertion that strong self-criticism might result in a high simultaneous occurrence with other physical symptoms. These findings may inform approaches for avoiding symptom complicating by addressing the behavior of both symptoms.

### Limitations

This study has several limitations. First, we showed networks of the BDI-II symptoms, which cannot be easily generalized to other rating scales for depression. Reportedly, network analysis is highly reproducible without assuming any latent variable. Further our findings were consistent with those of findings using the same scale and showed differences with those of a network study using a different scale. Second, our stability examination only supported part of the centralities, that is, strength. Caution should be applied when interpreting the findings regarding centrality. However, other network studies have failed to report sufficient CS-coefficients. Closeness and betweenness are often less stable than strength [34]. Accordingly, further research is warranted before confirming the instability of centrality measures. Finally, we should be concerned about the scarcity of sample size. Since this is a common limitation of network analyses, improvements through regularization have been proposed [28]. Although we used this method, a larger sample size could allow further improvements.

## Data Availability

Data supporting the findings of this study are available from the corresponding author upon reasonable request. Some of the data are not available since the subject has refused to disclose them. Restrictions in relation to potentially person identifiable information apply.
